# Renal dysfunction among adult HIV/AIDS patients on antiretroviral therapy at a tertiary facility in Ghana

**DOI:** 10.1186/s12882-018-1130-z

**Published:** 2018-11-21

**Authors:** Dorcas Obiri-Yeboah, Yaw Asante Awuku, Wonderful Alofa, Alice Charwudzi, Ebenezer Aniakwa-Bonsu, Evans Obboh, Paul Nsiah

**Affiliations:** 1 Department of Microbiology and Immunology, School of Medical Sciences, CoHAS, University of Cape Coast, Private Mail bag, Cape Coast, Ghana; 2 Department of Internal Medicine and Therapeutics, School of Medical Sciences, University of Cape Coast, Cape Coast, Ghana; 3 Department of Medical Laboratory Technology, School of Allied Sciences, University of Cape Coast, Cape Coast, Ghana; 4 Department of Haematology, School of Medical Sciences, University of Cape Coast, Cape Coast, Ghana; 5 Department of Chemical Pathology, School of Medical Sciences, University of Cape Coast, Cape Coast, Ghana

## Abstract

**Background:**

Kidney diseases have emerged as significant cause of morbidity and mortality in HIV subject on antiretroviral therapy (ART). In Ghana, routine follow up of HIV positive clients is by estimation of serum creatinine and urea levels. Glomerular Filtration Rate (GFR) is not routinely calculated and proteinuria is not routinely checked. This study sought to investigate the kidney profiles of adult HIV/AIDS patients being managed on ART at the Cape Coast Teaching Hospital (CCTH), Ghana.

**Methods:**

A hospital-based analytical cross sectional study with a retrospective component was conducted using systematic sampling method to recruit HIV/AIDS who visited the ART clinic. A total of 440 participants of both sexes aged 18 years and above, confirmed as HIV/AIDS positive and on ART were involved in this study. Blood and urine samples were collected from all subjects and the levels of serum creatinine and urea and proteinuria were estimated and eGFR calculated using the Modification of Diet in Renal Disease (MDRD) equations. Data analyses were performed using Stata version 13 software (Stata Corp, Texas USA).

**Results:**

The mean age (years) of participants was 45.5 years (±11.6) with 288 (65.4%) being on Tenofovir based ART regimen. The mean eGFR was found to decrease from 112.4 ml/min/1.73 m at baseline, to 103.4 ml/min/1.73 m after 6 months on ART and to a mean of 99.4 ml/min/1.73 m at recruitment into this study. Factors which were found to be associated with having eGFR < 60 included age, gender and CD4 count though not statistically significant. Patients > 45 years had the highest odds with OR 2.0 (95% CI: 0.8–5.1), females had higher odds with OR 1.5 (95% CI: 0.5–5.2), and those with CD4 count > 350 had OR of 0.4 (95% CI 0.2–1.3). A total of 30.9% of the participants had proteinuria at recruitment. TDF based ART regimen had no statistically significant effect on serum creatinine and urea levels.

**Conclusion:**

Estimated GFR decreased after 6 months among patients on ART despite normal serum creatinine and urea levels. This finding suggests that clients in care at HIV/ART clinics in Ghana may benefit from routine estimation of GFR and proteinuria.

**Electronic supplementary material:**

The online version of this article (10.1186/s12882-018-1130-z) contains supplementary material, which is available to authorized users.

## Background

The widespread implementation of combined antiretroviral therapy (ART) has resulted in a remarkable improvement in survival and decline in opportunistic infections globally [1]. The improvement in prognosis has changed the leading causes of morbidity and mortality in HIV infected patients from infectious to non-infectious causes [2] with kidney disease increasingly emerging as being significant. The renal dysfunction results directly or indirectly from a variety of mechanisms including the HIV infection, HIV-induced inflammation, comorbid diseases and drugs such as antiretroviral therapy [3, 4]. The nucleotide reverse transcriptase inhibitor, tenofovir disoproxil fumarate (TDF), has been found to causes a small but significant decrease in creatinine clearance in HIV/AIDS positive populations [5]. There has been increasing interest in TDF-associated tubular dysfunction (TD); the duration of TDF exposure appearing to play a part Cumulative TDF exposure is associated with increased risk of proximal tubular dysfunction in adults [6]. Recent publications attribute discontinuation of ART to increase HIV related mortality. This had necessitated recommendations for nephrotoxicity vigilance such as anticipation, early detection of risk factors through routine screening and implementation of preventative therapeutic strategies of ART induced kidney damage complications of long-term therapy and HIVAN [4, 7].

A recent study examining CKD in HIV-infected adult patients found a prevalence of 4.7% CKD; about 7% of the CKD population was ART-naïve [3]. A similar study in the Ashanti Region of Ghana reported a renal insufficiency prevalence of about 13 and 9% among HAART and HAART naïve patients respectively using the Cockcroft-Gault and the Modification of Diet in Renal Disease (MDRD) equation [8].

In Ghana the preferred first line regimen given to patients unless otherwise indicated is Tenofovir (TDF) combined with Lamivudine (3TC) or Emtricitabine (FTC) and Efavirenz (EFV) [9]. The increasing use of tenofovir across Sub-Saharan Africa requires further studies to address these questions [10]. There is very limited data available among patients receiving ART in Ghana. It is also noteworthy that routine follow up screening of HIV positive clients in Ghana mostly is by estimation of serum creatinine and urea. GFR is not routinely calculated and proteinuria is not routinely checked. The main aim of this study was to determine the serum concentration of creatinine, urea; urine protein and to grade the stage of the renal dysfunction among adult HIV/AIDS patients who are on ART based on GFR estimation at a tertiary facility in Ghana.

## Methods

### Study design and study population

This analytical cross-sectional study with some retrospective component was carried out at the antiretroviral (ART) clinic in a teaching hospital in the Central Region of Ghana. Participant recruitment was carried out between January to March 2017 using a systematic sampling method. Every third person (3:1) at the clinic on a particular day was given the opportunity to be part of the study using the arrangement of their folders for recruiting. If a selected person was found to be ineligible, the opportunity was given to the next folder in the queue. Eligible patients were people living with HIV/AIDS (PLHIV) aged 18 years and above, attending the ART clinic at the centre, and had been on ART for at least 6 months. Those with co morbid conditions such as hypertension, diabetes mellitus and kidney disease prior to the commencement of the ART were excluded. Retrospective data from the client’s clinic booklet were collected on their ART regimen, WHO clinical stage at recruitment and baseline laboratory data. A total of 440 eligible PLHIV visiting the ART clinic were recruited for this study. Additional information on study population, recruitment and sample size estimation is described in a sub study article [11]. The final sample size was estimated to target at least 50% of the 805 clients who were on ART at the end of 2016.

### Sample collection and analysis

Venous blood (5 ml) and single spot urine samples were collected in the morning from each patient on the day of scheduled follow-up. Serum samples were prepared by centrifugation (Nüve NF 200, Turkey) at 3000 rpm for 3 min. Creatinine and urea were analysed using automated Mindray Chemical analyser, BS-200E (Shenzhen, China). Urine samples were analysed for protein using urine dipstick test; URIT 2V^PG^ urine reagent strips containing tetrabromphenol blue 0.36 mg. The diagnosis of proteinuria was made based on the results of the urine dipstick colour by comparing with the corresponding colour blocks (> 30 mg/dl). Urine samples that were positive for blood, leucocyte and nitrate were excluded.

### Procedures

Socio-demographic characteristics like age, sex, level of education, occupation, religion, marital status were obtained using structured questionnaire (Additional file 1). The questionnaires were administered to the participants by research assistants trained by the principal investigator. Data on the period and type of ART was retrieved from the patient’s medical records. Other data extracted included the WHO clinical stage before initiating ART, duration on ART, ART regimen, baseline CD4 count, current CD4 count, plasma viral load after 6 months and if applicable (and available), after 1 year on ART were obtained from clinical records based on a data sharing agreement with the NACP. Data was entered into an Excel 2010 sheet anonymously.

### Estimation of glomerular filtration rate

The eGFR was calculated using the Modification of Diet in Renal Disease (MDRD) equations specific to non-IDMS calibrated creatinine was used. Renal dysfunction was graded from 0 to 5 according to the National Kidney Foundation guidelines.

### Statistical analysis

Data analyses were performed using Stata version 13 software (Stata Corp, Texas USA). Descriptive Statistics was performed and reported using frequency tables. Results were expressed as means with standard deviation or median with interquartile range. Unpaired t-test was used to compare mean values of continuous variables and independent sample t- test for categorical variables. Person’s correlation analysis was used to determine the association between the patient age, duration they have been on therapy and the clinical parameters. Bi-variable analysis of outcomes was carried out. For all statistical comparisons, a *P* < 0.05 was considered as statistically evidence of association.

## Results

### Participant characteristics

A total of 440 HIV positive individuals participated in this study. The mean age (years) of participants was (45.5 ± 11.6). The study participants have been described in a sub study analysis by Obiri-Yeboah et al [11]. Moreover, the median duration of HIV/AIDS diagnosis was 2 (IQR = 2–3) years. Majority of the participants (56%) were classified as WHO clinical stage 3 at the last staging before commencement of ART and as many as 288 (65.4%) are on Tenofovir based ART regimen (Table 1).

**Table 1:** Socio-demographic and other characteristics of 440 study participants

Variable	Mean (SD)/Median (IQR)/n (%)
Age (yrs.)	
Mean	45.5 (±11.6)
18–30	38 (8.6)
31–60	367 (83.4)
> 60	35 (8.0)
Gender	
Male	94 (21.4)
Female	346 (78.6)
Religion	
Christians	401 (91.1)
Moslems	34 (7.7)
Others	5 (1.1)
Marital status	
Single	100 (22.8)
Married/cohabiting	179 (40.8)
Divorced/widowed	160 (36.5)
Educational Status	
None to primary level	221 (50.2)
Up to secondary (senior high) level	196 (44.5)
Tertiary level	23 (5.2)
Employment	
Unemployed	67 (15.2)
Unskilled employment	346 (78.6)
Skilled employment	27 (6.1)
Place or Residence	
Rural	176 (40.0)
Urban	264 (60.0)
Duration of HIV diagnosis (years)	
Median	2 (2, 3)
< 2	83 (18.8)
2–5	172 (39.1)
> 5	185 (42.1)
Last WHO clinical stage before initiating ART	
1	21 (4.7)
2	101 (23.0)
3	249 (56.6)
4	69 (15.7)
ART regimen	
AZT + 3TC (FTC) + NVP	126 (28.6)
AZT + 3TC (FTC) + EFV	19 (4.3)
TDF + 3TC (FTC) + NVP	27 (6.1)
TDF + 3TC (FTC) + EFV	238 (54.1)
AZT + 3TC (FTC) + LPV/r	7 (1.6)
TDF + 3TC (FTC) + LPV/r	23 (5.2)

A higher percentage of the participants (90.2%) were using cotrimoxazole prophylaxis. The majority of the study subjects (65.7%) had been on ART for more than 2 years (> 2 years). The total number of participants with baseline CD4 count values available were 414 with mean of (215 ± 152.6) and the mean current CD4 count was (579.6 ± 203.0) of a total population of 396. A total of 149 (47.0%) of the participants had plasma viral load less than 1000 after 6 months on ART while 368 (89.6%) of participants had plasma viral load less than 1000 after 12 months on ART (Table 2).

**Table 2:** HIV associated clinical and laboratory characteristics of 440 study participants

Variable	Mean (SD)/Median (IQR)/n (%)
Use of Cotrimoxazole prophylaxis	
Yes	397 (90.2)
No	43 (9.8)
Duration on ART (years)	
< 1	82 (18.6)
1–2	69 (15.7)
> 2	289 (65.7)
Baseline CD4 count (*N* = 414)	
Mean	215.1 (±152.6)
< 200	197 (47.5)
200–349	159 (38.4)
350–500	42 (10.1)
> 500	16 (3.9)
Current CD4 count (*N* = 396)	
Mean	579.6 (±203.0)
< 200	9 (2.3)
200–349	33 (8.3)
350–500	94 (23.7)
> 500	260 (65.7)
Plasma viral load after 6 months on ART (*N* = 317)	
Median	1080 (198, 2598)
0	2 (0.6)
< 1000	147 (46.4)
≥ 1000	168 (53.0)
Plasma viral load after 12 months on ART (*N* = 411)	
Median	90 (20, 328)
0	85 (20.7)
< 1000	283 (68.9)
≥ 1000	43 (10.5)

### Renal function parameters

At baseline before starting ART, the proportion of clients with elevated serum urea levels (> 7.1 μmol/l) was 11.1% (*n* = 47). After 6 months on ART and at recruitment for this study, the proportion of clients with elevated serum urea levels were 12.3% (*n* = 48) and 4.6% (*n* = 18) respectively. For serum creatinine levels the proportions with elevated values (female: > 92 μmol/l; male: > 107 μmol/l were 3.1% (*n* = 13), 4.4% (*n* = 17) and 1.8% (*n* = 7) at baseline, 6 months on ART and at recruitment into this study respectively. A total of 20 (5.2%) participants were found to have a current eGFR values < 60 (ml/min/1.73 m) (Table 3).

**Table 3:** Renal function results of study participants at baseline, after 6 months on ART and then current

Variable	Mean (SD)//n (%)
Baseline blood urea levels, μmol/l (*N* = 425)	
Mean value	(4.5)
Normal values	378 (88.9)
High values	47 (11.1)
Blood urea after 6 months on ART, μmol/l (*N* = 391)	
Mean value	4.9 (±2.8)
Normal values	343 (87.7)
High values	48 (12.3)
Current blood urea values, μmol/l (*N* = 384)	
Mean value	3.7 (±2.5)
Normal values	369 (95.4)
High values	18 (4.6)
Baseline Creatinine levels, μmol/l (*N* = 425)	
Mean value	72.4 (±31.7)
Normal values	412 (96.9)
High values	13 (3.1)
Blood creatinine after 6 months on ART, μmol/l (*N* = 391)	
Mean value	75.9 (±25.3)
Normal values	373 (95.6)
High values	17 (4.4)
Current Creatinine levels, μmol/l (*N* = 387)	
Mean value	80 (±56.6)
Normal values	380 (98.2)
High values	7 (1.8)
Current eGRF(ml/min/1.73 m)	
≥ 90 (G1 = normal or high)	203 (52.5)
60–89 (G2 = mildly decreased)	164 (42.4)
45–59 (G3a = mildly to moderately decreased)	16 (4.1)
30–44 (G3b = moderately to severely decreased)	2 (0.5)
15–29 (G4 = severely decreased)	2 (0.5)
< 15 (G5 = kidney failure)	0 (0.0)

**Fig. 1:**
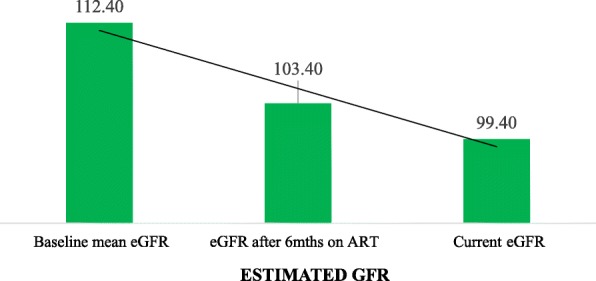
Distribution of mean eGFR of the study participants at baseline, after 6mths and currently at recruitment

**Fig. 2:**
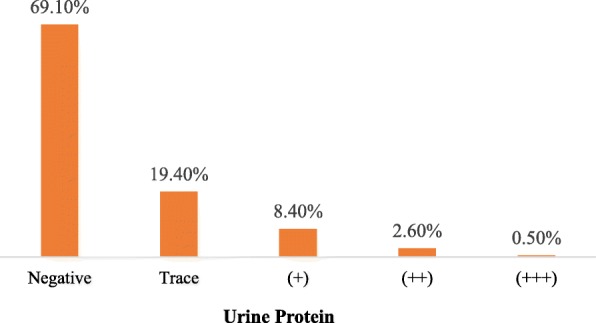
Distribution of urine protein of study participants

The baseline mean of eGFR of the study participants was 112.4(±42.8) whilst after 6 months on ART it was 103.4 (±38.5) and the current mean eGFR was (99.4 ± 35.1), Fig. 1. A higher percentage of the study clients (69.1%) had no urine protein detected while a total of 30.9% of the participants were positive (Fig. 2).

### Risk factor analysis

The study participants in age > 45 years had 2.0 (95% CI: 0.8–5.1) odds of having eGFR < 60 ml/min/1.73 m^2^ though there was no statistical evidence of association (*p* = 0.14). The odds for participants on a TDF based ART regimen was 1.1(95% CI: 0.4–2.4) and those with CD4 count > 350 cells/mm^3^ had 0.4 lower odds of having eGFR < 60 ml/min/1.73 m^2^ but these were all without statistical evidence of association (Table 4).

**Table 4:** Bivariate analysis for 20 participants with current eGFR < 60 ml/min/1.73 m^2^

Variable	eGFR < 60, {n (%)}	OR (95% CI)	*p*-value
Age (years)			
≤ 45	7 (35.0)	–	
> 45	13 (65.0)	2.0 (0.8–5.1)	0.14
Gender			
Male	3 (15.0)	–	
Female	17 (85.0)	1.5 (0.4–5.2)	0.54
*TDF based ART regimen			
No	7 (35.0)	–	
Yes	13 (65.0)	1.0 (0.4–2.4)	0.17
Duration of HIV Diagnosis (years)			
≤ 2	2 (10.0)	–	
> 2	18 (90.0)	2.1 (0.5–9.6)	0.30
Duration on ART (years)			
< 2	4 (20.0)	–	
≥ 2	16 (80.0)	1.2 (0.8–1.9)	0.14
Current CD4 count (cells/mm^3^)			
< 350	4 (21.0)	–	
≥ 350	15 (79.0)	0.4 (0.2–1.3)	0.13

## Discussion

The pathogenesis of CKD and its progression among PLHIV is multifactorial. This may include nephrotoxic antiretroviral medication, HIV viraemia, chronic systemic inflammation and the traditional cardiovascular risk factors such as Hypertension, Diabetes Mellitus, smoking [12, 13]. Our study examined the kidney profile of adult HIV/AIDS patients on ART and identified risk factors for CKD among PLHIV.

At recruitment into this study, 94.8% of study participants had eGFR > 60 ml/min/1.73 m, 5.2% had eGFR < 60 ml/min/1.73 m but none had eGFR < 15 ml/min/1.73 m, (end stage renal disease (ESRD) requiring renal replacement therapy). This pattern is a very good opportunity for education and intervention to slow down progression to ESRD which has cost and psychological implications for health care in Ghana. A large cohort of HIV study by Juega-Marino et al using MDRD estimation of eGFR and defining CKD as eGFR < 60 ml/min/1.73 m, showed an overall prevalence of 3.9–4.9% between 2008 and 2010 and about 87% with moderate CKD (stage 3) [12, 13]. This current study also used MDRD estimation and per the cut off used reported renal dysfunction prevalence of 5.2%. For PLHIV in sub-Saharan Africa varying prevalence of CKD have been reported ranging between 6 and 48% essentially due to variation in the diagnostic criteria used in the studies [14, 15]. A study in Ghana comparing ART naïve and clients on ART reported a higher rate of renal insufficiency among the clients on ART [8]. In a larger study among ART naïve clients in Ghana, Sarfo et al. concludes that renal dysfunction was very common among participants and was associated with increased risk of mortality [10] Studies done in other regions demonstrated a variable prevalence according to the social or sanitary level, clinical features, treatment and racial distribution [15, 16].

This study also showed progressive decline in mean eGFR over the period whilst on ART with baseline (ART naive) eGFR of 112.4 (±42.8), mean eGFR after 6 months on ART of 103.4 (±38.5) and at recruitment into this study of 99.4 (±35.1). In addition, proteinuria was detected in 30.9% of overall study participants (though majority was trace proteinuria) whilst only 1.8% had abnormal creatinine values. Routinely, ART clinics uses creatinine levels as a follow up test to identify CKD. Findings from this study suggests that creatinine values for detection of CKD is not a good practice since it can identify very late renal disease and miss early diagnosis, a crucial time where the natural history of the disease could be changed through specific interventions [17]. Creatinine clearance is influenced by many factors including diet, muscle bulk, medication and inter-current illness [15, 16]. This is therefore not an adequate test for screening PLHIV for CKD since it will give significant false negative results. Since most of the participants had advanced HIV disease (WHO stage 3 and 4) at initiation of ART, it is possible that decreased viremia after starting ART [11] improved their health and muscle mass and this could have affected creatinine values. Although better biomarkers for early detection of CKD especially in PLHIV are been worked on, eGFR and urine protein must be done in addition to creatinine clearance since eGFR has a higher sensitivity for early kidney disease [18, 19].

Seventy two point three percent (72.3%) of the study participants were in advance WHO stages (stages 3and 4) whilst 27.7% were in stages 1 and 2 at their last WHO clinical staging before initiating ART. Advance HIV disease is associated with higher prevalence of renal disease due to multiple postulated mechanisms [12, 14]. Identifying risk factors for CKD among PLHIV will guide practitioners in their care plan at the ART clinics. This study reports Tenofovir regimen was not associated with CKD among the clients. This could be that patients who had Tenofovir related kidney dysfunction were changed to other regimen and only those who tolerated were on that regimen. 65.4% (288) were on Tenofovir based ART regimen for this study and the odds of renal dysfunction tended to be higher among participants on TDF; however, this association did not reach the statistically significant level in this study. The adverse effect is reported commonly among low income communities, low CD4 count, advance disease and low body weight [14, 17]. The effect of Tenofovir is described as a double edged sword as there are many studies supporting our findings whilst others are on the contrary. In these studies some have demonstrated a decrease in GFR on Tenofovir regimen whilst others show normal GFR whilst on Tenofovir [20]. Tenofovir adverse effect has usually been reported as acute kidney injury [17] but sometimes seen as causing chronic kidney disease. Genetic factors and the histological subtype of kidney disease may show progression to chronic disease or resolve on ART initiation despite been a Tenofovir based regimen. Genetic factors including polymorphisms in APOL1 may have an important contribution to the burden of kidney disease as shown by other studies [21, 22]. Older age is a known risk factor for CKD in HIV positive and negative populations. Worth noting also is the phenomenon of “premature ageing” as described in the HIV population as a result of the immune dysregulation [13]. Screening and early diagnosis to identify early disease must be adhered to in ART clinics at all levels in Ghana so as to initiate early therapy and change the natural history of renal disease among PLHIV.

Limitations of this study include the fact that the weight of participants was not monitored in the study period which would have provided useful information in interpreting their creatinine values. A larger longitudinal study is needed to properly evaluate the effect of HIV and ART on the kidney of function PLHIV in Ghana.

## Conclusion

The prevalence of renal dysfunction among the HIV positive cohort on ART in this study is significant and yet most would have been missed if serum creatinine level is used as the screening modality. Screening and early diagnosis using eGFR and urine protein might be useful at all levels of ART clinics in Ghana.

## Additional file

Additional file 1: Questionnaire. (DOCX 41 kb)

## Abbreviations

AIDS: Acquired Immunodeficiency syndrome
ART: Antiretroviral therapy
CCTH: Cape Coast Teaching Hospital
CD4: Cluster of differentiation
CI: Confidence interval
CRD: Chronic kidney disease
CRF: Chronic kidney failure
ESRD: End-stage renal disease
GFR: Glomerular filtration rate
GHS: Ghana health service
HIVAN: HIV-associated nephropathy
NACP: National AIDS control programme
NNRTIs: Non-nucleoside reverse transcriptase inhibitors
NRTIs: Nucleoside reverse transcriptase inhibitors
PIs: Protease inhibitors
UCC: University of Cape Coast
WHO: World Health Organization
