# Supplementary material for: Renal dysfunction among adult HIV/AIDS patients on antiretroviral therapy at a tertiary facility in Ghana
Source: BMC Nephrol. 2018 Nov 21;19:333. doi: 10.1186/s12882-018-1130-z (PMC6249759; doi:10.1186/s12882-018-1130-z)
Supplement: Supplementary file 1 — Questionnaire. (DOCX 41 kb) [file 12882_2018_1130_MOESM1_ESM.docx]

**APPENDIX A: QUESTIONNNAIRE**

| **DATE:** | | **STUDY CODE *(pre-printed)*:** | | **UNIQUE NUMBER:** |
| --- | --- | --- | --- | --- |
| **NAME OF INTERVIEWER:** | | **SIGNATURE:** | |  |
| **WEIGHT (Kg):** | | **HEIGHT (cm):** | |  |
| **SOCIO-DEMOGRAPHICS** | | | | |
| **No.** | **QUESTIONS** | **CODE** | | **RESPONSE(S)** |
| **Q1** | **AGE** | **(*write the actual number down)*** | |  |
|  | **GENDER** | 1. **Male** 2. **Female** | |  |
| **Q2** | **OCCUPATION** | 1. **Skilled employment** 2. **Unskilled employment** 3. **Unemployed** 4. **Other** | |  |
| **Q3** | **RELIGION** | 1. **Christian** 2. **Moslem** 3. **Others** | |  |
| **Q4** | **RESIDENCE** | 1. **Rural** 2. **Urban** | |  |
| **Q5** | **MARITAL STATUS** | 1. **Single** 2. **Married** 3. **Cohabiting** 4. **Divorced** 5. **Widowed** | |  |
| **Q6** | **LEVEL OF EDUCATION** | 1. **No formal education** 2. **Primary** 3. **J.S.S** 4. **Middle school form 4** 5. **Secondary** 6. **Tertiary** | |  |
| **Q7** | **HOW LONG HAS IT BEEN SINCE YOU WERE DIAGNOSED?** | **(*write the duration as much as possible in months)*** |  | |
| **Q8** | **DO YOU CURRENTLY SMOKE** | 1. **Yes** 2. **No** |  | |
| **Q9** | **HISTORY OF TB SINCE HIV DIAGNOSIS** | 1. **Yes** 2. **No** |  | |
| **FROM CLIENT BOOKLET** | | | | |
| **Q10** | **LAST WHO CLINICAL STAGE** | **(*Write in 1,2,3,4)*** | | **Date done:**  **__ / __/ __** |
| **Q11** | **IS CLIENT ON SEPTRIN?** | 1. **Yes** 2. **No** | |  |
| **Q12** | **ART REGIMEN** | 1. ***Zidovudine +* Lamivudine (or FTC) + Nevirapine** 2. ***Zidovudine +* Lamivudine (or FTC) + Efavirenz** 3. ***Tenofovir +* Lamivudine (or FTC) + Nevirapine** 4. ***Tenofovir +* Lamivudine (or FTC) + Efavirenz** 5. ***Zidovudine +* Lamivudine (or FTC) + Lopinavir/r** 6. ***Tenofovir +* Lamivudine (or FTC) + Lopinavir/r** | |  |
| **Q13** | **WHEN WAS ART STARTED?** | 1. **6mths -<1 year** 2. **1-2 years** 3. **>2 years** | |  |
| ***THANK YOU*** | | | | |
